# A novel method intersecting three-dimensional motion capture and medial elbow strength dynamometry to assess elbow injury risk in baseball pitchers

**DOI:** 10.1038/s41598-023-39504-9

**Published:** 2023-07-28

**Authors:** Toshimasa Yanai, Kengo Onuma, Ryan L. Crotin, Daisuke Monda

**Affiliations:** 1 Research Institute of Baseball Science and Faculty of Sport Sciences, Waseda University, 2-579-15 Mikajima, Tokorozawa, 359-1192 Japan; 2 Graduate School of Sport Sciences, Waseda University, Tokorozawa, Japan; 3 Human Performance Laboratories, Louisiana Tech University, Ruston, LA USA; 4 Sports Performance Research Institute New Zealand, Auckland University of Technology, Auckland, New Zealand; 5 Saitama Seibu Lions, Tokorozawa, Japan

**Keywords:** Biophysics, Health care, Risk factors, Engineering

## Abstract

In baseball pitching, resultant elbow varus torque reaches the peak value of 50–120 N m, exceeding the joint failure limit that risks damage to the ulnar collateral ligament (UCL). In-vivo methodology is lacking to assess whether pitchers have sufficient muscular strength to shield UCL and how strongly the elbow musculature must contract to minimize valgus loading on UCL. This study introduces a method to assess relative percentages of muscular varus strength required to unload the UCL. The maximum voluntary isometric varus strength (MVIVS) produced by the medial elbow musculature and the maximum resultant varus torques at elbow in pitching fastballs and other types were measured for two professional pitchers. Simulation was conducted to determine the relative percentages of MVIVS required to unload the UCL to varying degrees and the impact of athletes’ previous UCL reconstruction on the relative percentages was examined. The maximum resultant varus torque in pitching was found to range 72–97%MVIVS depending on the type of pitch. The elbow musculature had to produce 21–49%MVIVS to avoid acute failure of intact UCL whereas the corresponding requirements were 39–63%MVIVS for UCL reconstructed joint. The method offers new insight into baseball pitcher’s training/rehabilitation and physical assessment to reduce the risk of UCL injury.

## Introduction

The medial aspect of elbow joint is one of the most common sites of overuse injuries to baseball pitchers^1^. According to the literature on MLB pitchers, elbow injuries accounted for approximately 30% of all baseball related injuries^2^ and the so called “Tommy John surgery,” a surgical protocol for reconstructing damaged ulnar collateral ligament (UCL)^3^, has been performed more frequently in the present decade^4–6^. Medical literature^7–12^ suggested greater ball velocity, excessive pitching counts, fatigue, improper conditioning, and, although debatable in biomechanical terms^13^, throwing too many breaking balls too young are all considered elbow injury risk factors. The mechanism of elbow injury behind these risk factors is the UCL’s inability to withstand repeated valgus stress which opens the inner elbow compartment^14^ and causes elongation of the ligament that overtime can lead to attenuation and failure^15^.

**Figure 1:**
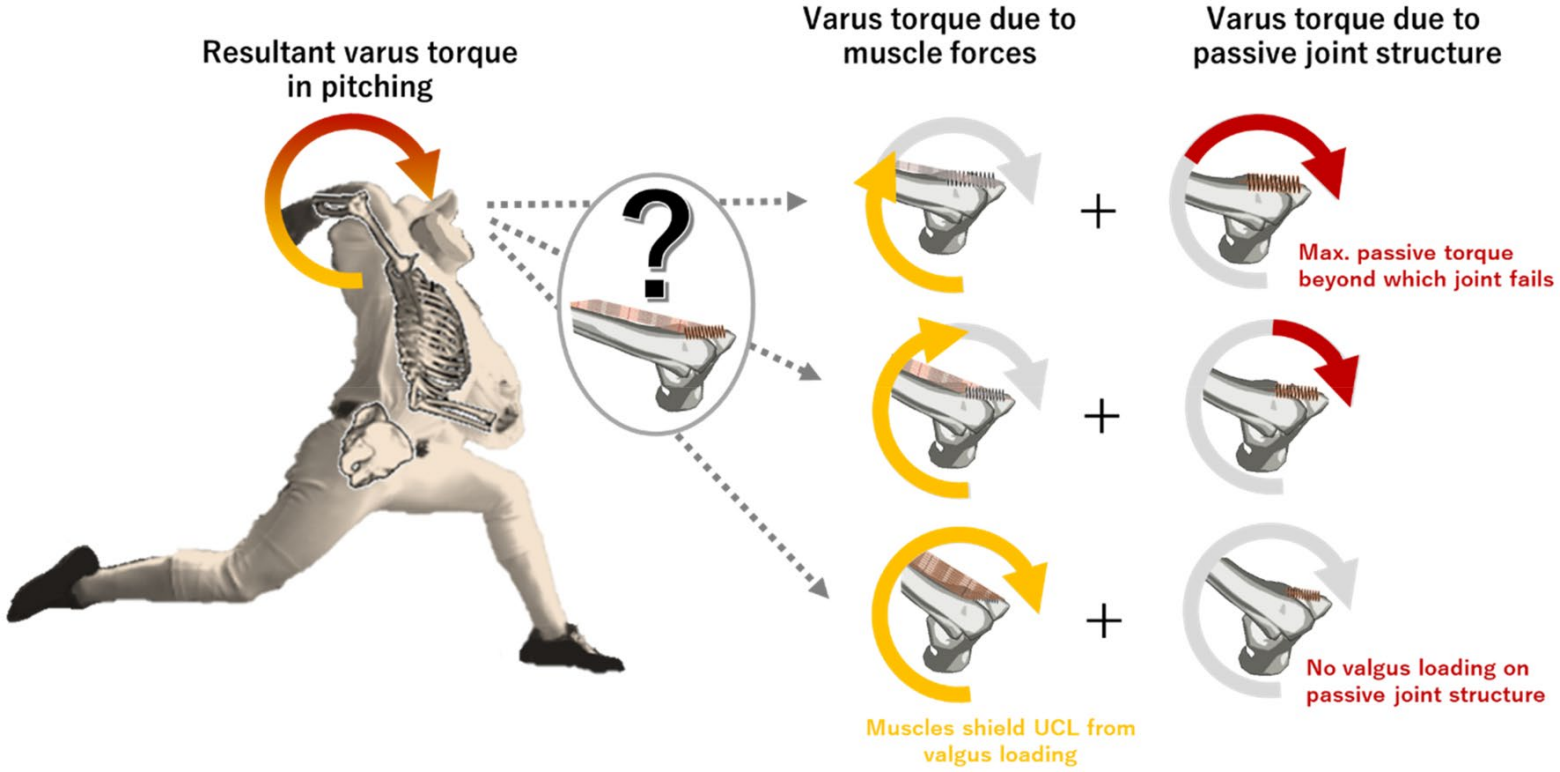
Schematic representation of elbow varus torque. Elbow varus torque is produced by contractile force of medial elbow musculature and passive forces due to tension and compression of joint structures (UCL, joint capsule, articulation, etc.). It is not yet known whether baseball pitchers’ elbow musculature is strong enough to eliminate valgus loading on the UCL and how strongly the musculature must contract to eliminate valgus loading on UCL or to reduce the loading to fall within the safe range. *Note*: The pitcher’s image was created by the author (TY) from a video clip of a pitcher and the corresponding skeletal image of his pitching motion visualized by the custom software JointMotionViewer 1.0.

The anterior bundle of the UCL is the primary stabilizer to valgus loading^14,16^. Cadaveric studies demonstrated that the valgus load applied to a flexed arm is resisted by varus torque that opposes medial elbow opening and is generated primarily by the UCL, joint articulation, and joint capsule up to the joint failure load of about 35 N m^17,18^. In baseball pitching, elbow varus torque reaches the peak value of 50–120 N m near the end of cocking phase^19–28^ that is beyond the failure limit of the elbow joint. Therefore, contractile force of the muscles surrounding the medial aspect of elbow joint is necessary to reduce the amount of loading on the UCL with every pitch and to prevent joint failure from occurring in competitive baseball pitchers (Fig. 1).

Cadaveric studies have indicated that the medial elbow musculature such as flexor carpi ulnaris, flexor digitorum superficialis, and pronator teres need to coordinate medial elbow compression that reflects elevated varus torques to reduce the loading on the UCL^29–35^. The amount of varus torque that the elbow musculature can generate was estimated to be 35 N m by a computational modeling analysis approach^36^. A forward dynamics simulation of the pitching delivery^37^ estimated that the elbow musculature should contribute to 35–57% of the maximum resultant varus torque (i.e., 40–65 N m of the 115 N m resultant torque) to resist the peak valgus load imposed by the pitching motion. Although the estimated muscular contribution is substantial, the remaining amount of varus torque which is supposedly produced by the passive joint structure such as UCL and joint capsule is likely to exceed the joint failure load. There seems to be some disagreement among the kinetic outcome of pitching motion analysis, computational modeling analysis of muscle strength, and the structural strength of the elbow joint.

One missing piece of the puzzle is the in-vivo knowledge of baseball pitcher’s muscular varus strength, such as whether baseball pitchers’ elbow musculature is capable of functioning as the stress-shielding contributor to resist the valgus loading to unload the UCL and how strongly the musculature must contract to reduce the loading to fall within the safe range (i.e., the rage below the yielding point of the stress–strain relation of UCL). Given the volume of pitch that each pitcher throws regularly in a daily practice or a game situation, the muscular varus torque executed in every pitch should be substantially lower than the maximum varus torque producible by the elbow musculature of the pitcher. Mechanical relations among the muscular varus strength, the relative intensity of muscular output required for pitching, the structural strength of passive joint structure, and pitch count needs to be explored in-vivo to advance the knowledge of elbow injury mechanism in baseball pitchers. The purpose of this study was to introduce an in-vivo biomechanical assessment procedure for determining relative percentages of muscular varus strength required to unload the UCL to varying degrees, ranging from the degree to which the joint with reconstructed UCL would fail to the degree to which UCL could be completely unloaded, for baseball pitchers, and to discuss the implications of the analysis outcome for training and injury prevention.

## Methods

Two professional baseball pitchers (ages: 26 ± 1.4 years-old, mass: 83 ± 7.8 kg, height: 1.79 ± 0.014 m) were analyzed in this study. Both participants had just returned to pitching in simulated game situations after UCL reconstruction surgery, but they were self-declared as healthy, having no physical injury or pain on the day that hindered their normal pitching performance. The study procedure was approved by the Ethics Review Committee on Research with Human Subjects of Waseda University and the risks and benefits associated with voluntary participation were explained to each participant, and written acknowledgement of the informed consent was obtained from participants and all methods were carried out in accordance with the standards of the Declaration of Helsinki.

**Figure 2:**
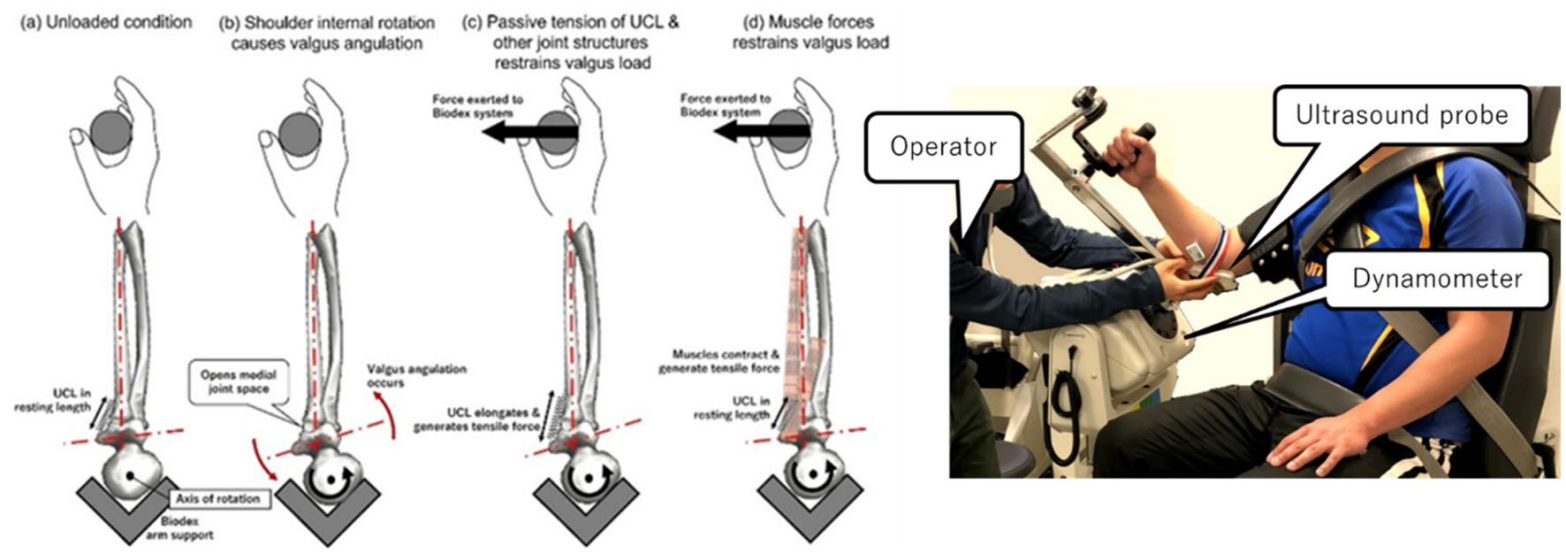
Mechanical relations among shoulder internal rotation torque, valgus angulation, and varus torques produced by passive constraints (e.g., UCL, joint capsule, and body articulation) and the elbow musculature of the participant positioned on Biodex system.

**Figure 3:**
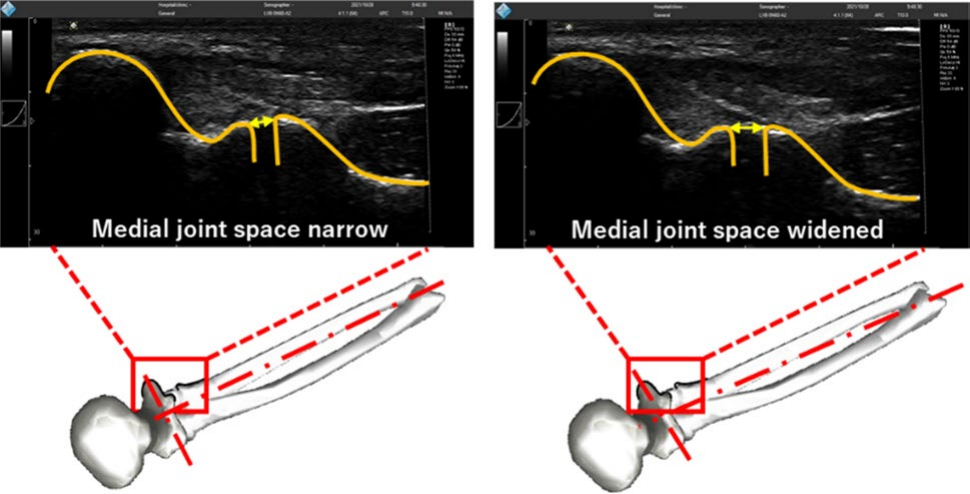
Ultrasound images of medial joint space. The distance between the trochlea of the humerus to the sublime tubercle of the ulna (indicated by arrows) was measured during assessment. Increasing distance indicates the UCL and joint capsule on the ulnar side are elongated and produces tensile forces to resist valgus loading.

The data collection consisted of two parts. The first was assessment of maximum voluntary isometric strength in generating elbow varus torque. Second, motion capture of the pitching delivery was enacted to compute the maximum joint resultant varus torque at elbow generated during pitching delivery. The data collection sessions were conducted in two separate days. On day 1, a Biodex System 4 (Biodex Medical Systems, NY, USA) and an ultrasound device (ArtUs EXT-1H; TELEMED Ultrasound Medical Systems, Vilnius, Lithuania) were used to measure the maximum voluntary isometric strength with a recently developed method. ^38^ In short, the method resembles the typical procedure used to measure the strength of shoulder internal rotator muscles in the modified neutral position (Fig. 2). In this position, the valgus-varus axis of the elbow joint coincided with the internal–external rotation axis of the shoulder joint (Fig. 2a), and the shoulder internal rotation torque measured with the Biodex system could represent the net varus torque generated by elbow musculature and the passive constraints including UCL, joint capsule, and bony articulation. Each participant was fastened to the system and asked to perform ramp contraction up to 100% of his maximum voluntary isometric strength of shoulder internal rotators while maintaining the maximal voluntary isometric contraction of the elbow varus stabilizers, such as flexor carpi ulnaris, flexor digitorum superficialis, and pronator teres^29–35^. The ultrasound device manually placed along the anterior bundle of the UCL was used to monitor the opening of the medial joint space between the trochlea of the humerus to the sublime tubercle of the ulna during assessment (Fig. 3). An opening of the medial joint space (i.e., increasing distance between the two bony landmarks) indicates an increase in valgus angulation of the elbow joint (Fig. 2b), which causes the UCL and joint capsule on the ulnar side to elongate and the humeroradial joint to be compressed. The tensile forces generated in the UCL and joint capsule and the compressive force generated at the humeroradial joint produce a varus torque that functions as a passive restraint to the valgus loading (Fig. 2c). In contrast, the torque measured with the Biodex system represents the elbow muscular varus torque if the medial joint space does not open (Fig. 2d). In this assessment, the largest torque recorded while the medial joint space was narrower than an individualized threshold distance was determined as the varus strength of the participant. The threshold distance was predetermined for each participant through a valgus stress test: In a supine position with the shoulder joint externally rotated and abducted to 90°, the elbow joint flexed to 90°, and the elbow muscles relaxed completely, a 0.5-kg weight was applied at the wrist, which, together with the weights of the hand and forearm, produced the total valgus load of approximately 10–15% of the joint failure load of about 35 N m^17,18^. A video analysis software (Frame-DIAS V, Q’sfix, Tokyo, Japan) was used to measure the distance between the two bony landmarks for the ultrasound images recorded during the strength assessment as well as the valgus stress test. After a few submaximal trials of familiarization, the strength assessment was repeated twice with a 2–3-min rest interval. Additional assessment was to be performed if the torque measured on the second attempt was increased by 10% or more over the first attempt, but no additional assessment was required for the participants (the increase was < 1 N m for both participants). The largest torque over the trials was taken as the maximum voluntary isometric varus strength (MVIVS) of the participant’s elbow.

On day 2, the data collection for pitching performance was conducted in the bullpen of the team’s indoor baseball facility. After a self-determined warm up session (jogging, stretch, shoulder exercises, play catch etc.), each participant threw 4–6 pitches each for all types of pitches that they used in regular games (Sub1: 4 pitches each of fastball, slider, curveball, sinker & Sub2: 6 pitches each of fastball, slider, curveball, cut ball, changeup) in their game effort. A Rapsodo system (Pitching 2.0, Rapsodo LLC) was used to measure the ball speed of each pitch. Pitching delivery motions were recorded with an electromagnetic tracking device^39^ (Liberty, Polhemus, USA) which measured the six degrees of freedom of motion for two sensors attached to the participant’s body at the nominal sampling rate of 240 Hz. A system sensor was attached on the skin over the dorsal surface of the distal 1/5th of the forearm of the dominant side with double-sided tape and elastic tape and another sensor was attached to the upper arm with a plastic cuff that wrapped around the upper arm to minimize the influence of skin artifacts. The cables of the sensors were bundled together and held by an operator to prevent the cabled from interfering the pitching motion.

An inverse dynamics approach determined the valgus load imposed at elbow in each pitch. For this analysis, the raw position data were smoothed with a fourth-order Butterworth filter at 15 Hz and each body segment was modelled as a rigid body having the inertial parameters for Japanese athletes^40^. The hand and the ball were assumed to move together with the forearm so that a system of ball, hand and forearm was considered as a rigid body, as in the literature^41^. The computed joint resultant torque was decomposed into three orthogonal components: flexion torque, pronation torque, and varus torque. The varus torque is the net torque exerted on the forearm by the muscles, ligaments and other joint structures that connect the forearm and arm at the elbow joint in response to the valgus load generated by pitching motion. For each pitch type of each pitcher, the mean value of all recorded pitches was computed for subsequent analysis.

The maximum varus torque attained during pitching delivery was expressed as a ratio to the MVIVS (%MVIVS) so that the relative intensity of muscular output required to resist the valgus load completely to unload the UCL could be indicated. In addition, the minimum relative intensity of muscular output required during pitching to reduce the valgus load on the UCL and other passive structure to fall within the safe range of the elbow structure was estimated, as follows:

$$\% MVIVS_{min} = \frac{maximum\, varus\, torque\, in\, pitching - allowable\, joint\, load}{{MVIVS}} \times 100$$

where allowable joint load is the amount of valgus torque that the passive joint constraints should be able withstand safely (in return, its reaction is the varus torque that the passive joint constraints could produce to maintain the joint integrity).

The logic behind this formula is as follows: The varus torque at elbow joint in pitching must be produced by an active torque generated by elbow musculature and a constraint torque produced by passive joint structure such as UCL and joint capsule. Because the constraint torque has an upper limit due to the structural strength of the joint, the surplus torque needs to be generated by the elbow musculature. Therefore, the computed value of %MVIVS_min_ was indicative of the theoretical intensity of muscular contractile output required to minimize the effects of valgus loading on the integrity of passive joint structure during pitching. The allowable joint load was estimated to be 20 N m (the mean of 16–24 N m) for reconstructed elbow and 28 N m for healthy elbow, based on (a) the joint failure load (the maximum valgus load that an elbow joint could withstand before failure) reported in cadaveric studies was approximately 20–30 N m for UCL reconstructed elbows and 35 N m for elbows with intact UCL^17,18^, and (b) the linear region of the type I collagen tissue reported by a tissue-mechanics study^42^ was 20–80% of the ultimate strength. All measured and computed variables were presented as means and standard deviations.

## Results

**Figure 4:**
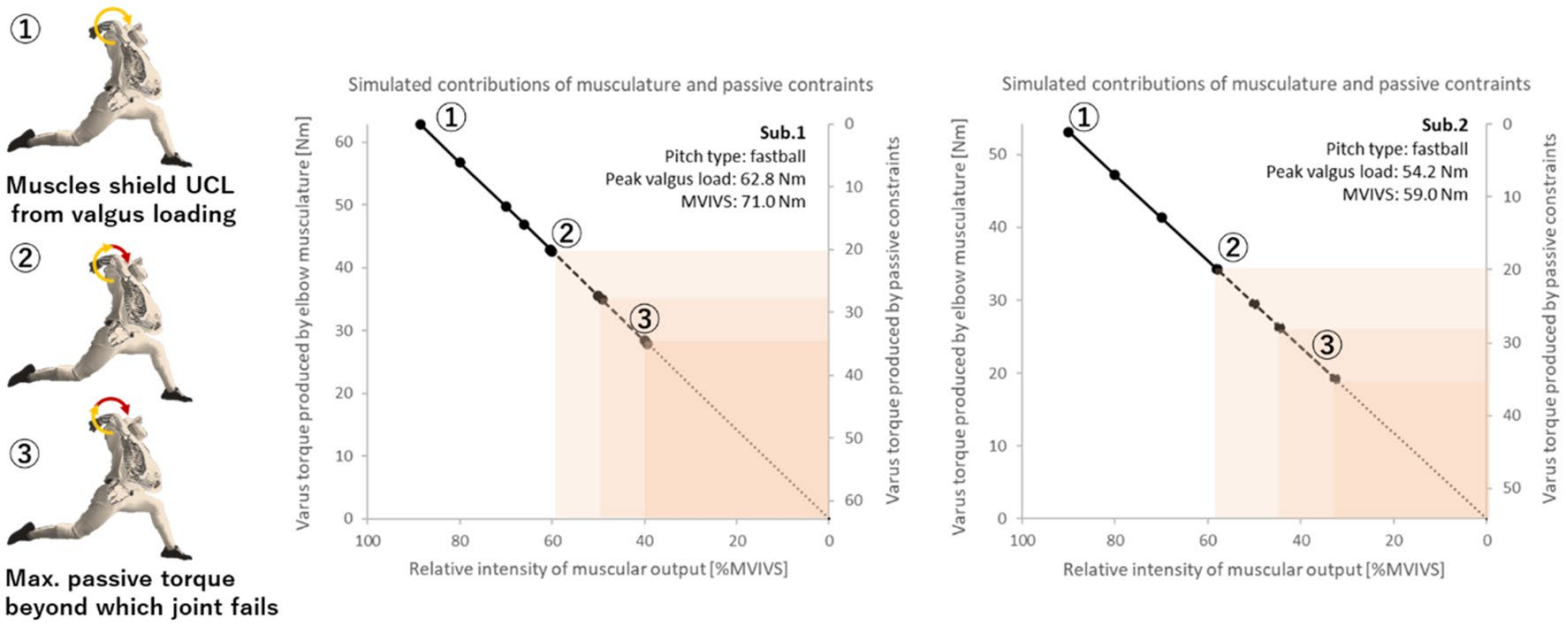
Simulated contributions of the varus torque produced by the elbow musculature and the passive joint constraints such as UCL, joint capsule, and articulation to the peak varus torque in fastball pitching of two participants. As the joint failure load is reported to be 20–30 N m for UCL reconstructed elbows and 35 N m for healthy elbows with intact UCL^17,18^, and the linear region of the type I collagen tissue reported by a tissue-mechanics study^42^ was 20–80% of the ultimate strength, the allowable joint load was estimated to be 20 N m (the mean of 16–24 N m) for reconstructed elbow and 28 N m for healthy elbow. The elbow musculature needs to generate a contractile torque with a relative intensity of approximately 60%MVIVS to reduce the valgus load on the passive structure to fall within the safe range of the elbow joint at or around maximal external shoulder rotation near the end of cocking phase in fastball pitching. *Note*: This simulation was conducted with an assumption that the elbow musculature involved in varus torque production responded similarly to load stimuli in the MVIVS assessment for measuring 1 RM and in the pitching delivery.

The MVIVS of the elbow dynamic stabilizers were 71 N m for Sub1 and 59 N m for Sub2. The maximum varus torque produced in pitching was attained immediately before the maximum shoulder external rotation angle was reached for all pitch types thrown by the participants. At this instant, the elbow flexion angle (0° at full extension) was 87.3 ± 3.3° for Sub1 and 88.4 ± 5.1° for Sub2. The magnitudes of maximum resultant varus torque ranged from 51.5 ± 1.6 N m for curveballs (ball velocity = 30.8 ± 0.4 m/s) to 62.9 ± 2.7 N m for fastballs (ball velocity = 38.1 ± 0.4 m/s) for Sub1, accounting for 72.5–88.6%MVIVS of his elbow dynamic stabilizers (Table 1). For Sub2, the peak valgus load ranged from 43.1 ± 3.8 N m for changeups (ball velocity = 32.1 ± 0.7 m/s) to 57.3 ± 11.2 N m for slider (ball velocity = 33.0 ± 0.6 m/s), accounting for 73.1–97.1%MVIVS of his dynamic stabilizers. The minimum muscular contractile output required to reduce the valgus load on the passive structure to fall within the safe range was 44–60%MVIVS_min_ for Sub1 and 39–63%MVIVS_min_ for Sub2, whereas the corresponding requirements were reduced below 50%MVIVS_min_ if the participants had intact UCL (Fig. 4).

**Table 1:** Pitch velocity and valgus load imposed during pitching.

Subject	Pitch type	Velocity [m/s]	Max varus torque [N m]	%MVIVS	%MVIVS_min_ Reconstructed	%MVIVS_min_ Intact
1	Fastball	38.1 ± 0.4	62.9 ± 2.7	88.6	60.4	49.2
	Slider	33.3 ± 0.2	58.3 ± 2.3	82.1	53.9	42.7
	Curveball	30.8 ± 0.4	51.5 ± 1.6	72.5	44.4	33.1
	Sinker	32.2 ± 0.3	53.9 ± 1.9	75.9	47.7	36.5
2	Fastball	36.4 ± 0.4	54.2 ± 5.6	91.9	58.0	36.9
	Slider	33.0 ± 0.6	57.3 ± 11.2	97.1	63.2	41.3
	Curveball	30.0 ± 0.6	55.0 ± 11.0	93.2	59.3	38.0
	Cut ball	35.9 ± 0.5	55.7 ± 7.5	94.4	60.5	39.0
	Changeup	32.1 ± 0.7	43.1 ± 3.8	73.1	39.2	21.3

Velocity in m/s represents ball velocity for each pitch type. Max varus torque represents the peak elbow varus torque recorded during the pitching delivery.

%MVIVS; Percentage of the Max varus torque in pitching with respect to the maximum voluntary isometric varus strength (MVIVS).

%MVIVS_min_; The minimum percentage of the maximum voluntary isometric varus strength required to reduce the valgus load on the passive structure to fall within the safe range of the elbow joint with reconstructed UCL and intact UCL.

## Discussion

The method combined the dynamometry and motion capture technology to evaluate if baseball pitchers have sufficient muscular varus strength to withstand the valgus loading and how strongly the musculature must contract to reduce the loading on the UCL to fall within safe range. For the two participants to completely shield the valgus loading from the UCL during pitching, the elbow musculature had to produce a varus torque at 72.5–97.1%MVIVS in every pitch. Based on a typical exercise intensity in weight training for repeating 15–25 repetitions per set ranges from 40 to 65% of 1RM^43^, the relative intensity of elbow muscular contractile output is expected to be in a similar range for a typical pitcher who throws 15–25 pitches per inning. The demands on the elbow musculature to generate a varus torque repeatedly at the intensity of 72.5–97.1%MVIVS seems unlikely from the viewpoint of the muscle’s force generation capability. The results suggest that the elbow musculature of these participants is not likely to have produced the entire varus torque required to completely shield the UCL from the peak valgus load in pitching although the participants’ muscular varus strength exceeded the varus torque required for pitching. The method estimated 39–63%MVIVS_min_ of varus torque is required for the participants to minimize the effects of valgus loading on the integrity of passive joint structure during pitching (Table 1). In the context of typical weight training, a training load of 45–50%1RM intensity allows the lifter to continue 20–30 repetitions^43^. This suggests that the intensity is submaximal and in a reasonable range to throw balls repeatedly in each inning. The relative intensity of muscular output determined from this method should provide a new insight into baseball pitcher’s strength requirement for reducing the risk of UCL injury in baseball pitchers.

**Figure 5:**
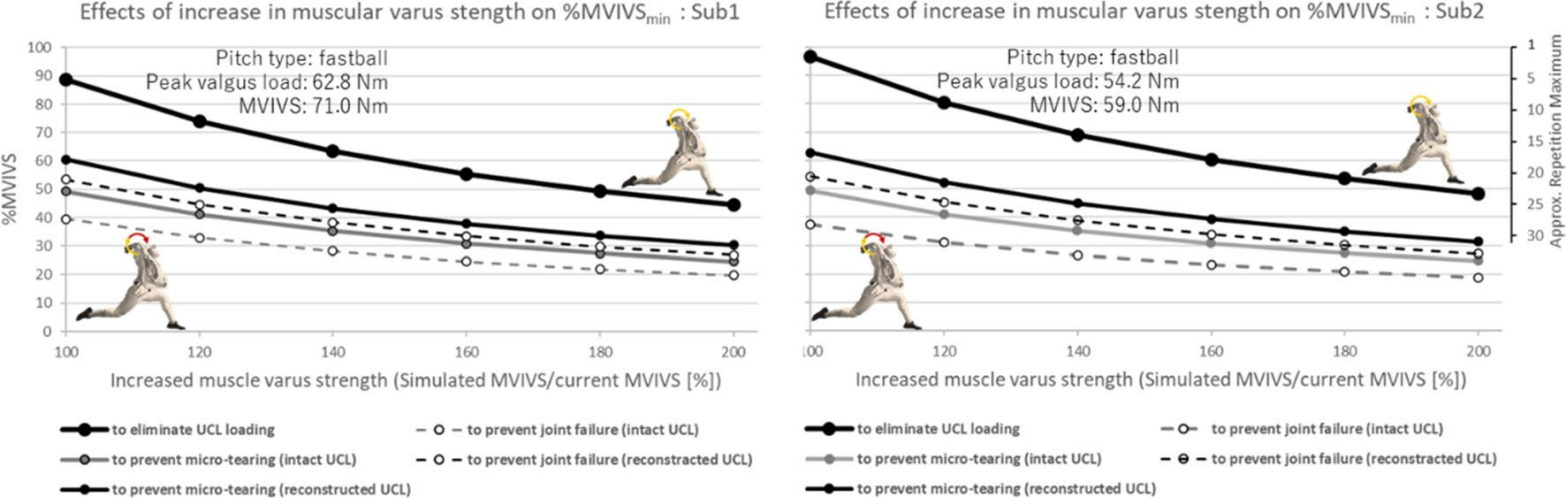
Estimated effect of increased muscle varus strength on the relative intensity of muscular output required to unload UCL to varying degrees. The abscissa indicates simulated maximum muscular strengths of the pitcher expressed as the ratio of increased MVIVS relative to the current MVIVS and the ordinate indicates the relative intensity of muscular output required (**a**) to eliminate valgus loading on UCL, (**b**) to produce a varus torque to reduce the valgus load on the passive structure to fall within the safe range of the elbow joint (thick solid lines), and (**c**) to prevent joint failure (dashed lines).

The impact of previous UCL injury and its reconstruction might be illustrated in the difference in the muscular output required to avoid UCL failure. If the participants had the natural elbow joint with intact UCL and the joint failure load were higher (35 N m), the minimum strength demands on the elbow musculature to reduce the loading on the UCL to fall within safe range should have been lower (< 50%MVIVS, Table 1) than what had been calculated post-operatively (39–63%MVIVS). This simulation illustrates clearly that the required muscular output for preventing UCL reinjury increases by 10–20%MVIVS post-operatively. On the other hand, the required muscular output reduces systematically if the participants increase their muscular varus strength (Fig. 5). This comparative simulation analysis indicates the importance of promoting both their strength and muscle endurance of the medial elbow musculature for pitchers who had UCL reconstruction surgery, in order to reduce the risk of reinjury of the weakened UCL.

The methodological procedure presented in this study is generalizable whereas the quantitative data are not. Therefore, the discussion of the quantitative results should remain descriptive in nature although the outcomes of the extended theoretical analysis provide unique clinical implications beyond the cases. In addition, the present study was not designed to identify the specific muscles that produced the maximum voluntary isometric varus torque or the resultant varus torque in pitching delivery, so that a complete partitioning of the resultant varus torque in pitching into active and passive elements is beyond the scope of the study. Another limitation is that the measurement system used to determine the varus torque in pitching is different from most other studies. Similarly to a film-based analysis^23,41^ in which manual digitizing of body landmarks introduces a human error into measurement and a camera-based real-time motion capture system in which reflective markers attached to the skin of the participants introduces measurement error due to skin-artifacts^44,45^, the present analysis involving electromagnetic goniometry introduces a measurement error due to skin-artifacts^46,47^. As the influences of these errors on the biomechanical variables are generally minimized by applying a digital filter and correcting joint position of adjacent segments, the same procedure was applied in this study. As a result, the peak valgus load in fastball pitching presented in this study (54–63 N m) and additional values calculated from 92 healthy baseball pitchers (collegiate, semi-professional, and professional pitchers) who threw fastballs (72.5 ± 18.3 N m) fell in the range of the corresponding values reported from similar populations^19–28^. Agreement was reached concerning the numerical results obtained in the present study with existing literature, therefore the present data collection method and the obtained results for the kinetic analysis of pitching motion are valid.

The major limitation of this study is that the muscle varus strength was measured in an isometric condition at a nominal elbow flexion angle of 90° with the forearm in neutral pronation-supination, so that the measured maximum voluntary varus torque may be either an underestimation or an overestimation of the maximum torque in the actual contraction condition of the involved muscles during the pitching delivery due to the length- and velocity-dependence of muscle strength. Healthy elbow joints have very limited valgus-varus range of motion and the potential effect of valgus or varus joint motion on the muscle contraction condition should be minimal. However, joint motion in flexion–extension and pronation-supination is expected to make unignorable effects on the muscle contraction condition of the elbow flexor-pronator muscle group. At the instant that the varus torque reached the peak value near the end of the cocking phase, the participants’ elbow joints were flexed slightly more than 90° (Sub1: 94.7 ± 3.1° & Sub2: 95.9 ± 11.0°) and mostly supinated (Sub1: − 5 ~ 42° & Sub2: 24–48°) while undergoing extension (Sub1: 2051 ± 94°/s & Sub2: 2382 ± 437°/s) with axial forearm rotation in either directions depending on the pitch type (ranged from pronating at 289°/s to supinating at 184°/s for Sub1 & pronating at 101°/s to supinating at 293°/s for Sub2). Cadaveric study reported that the elbow flexor-pronator muscles such as flexor carpi ulnaris, flexor digitorum superficialis, and pronator teres have varus moment arms and, with the exception of flexor carpi ulnaris, have flexor moment arms^29,48^. The difference in the length of the muscle–tendon complex due to the difference in joint configurations between the pitching condition and the strength testing condition was estimated as the product of the difference in the joint angle and the reported moment arms^29,48^. It was less than 10 mm for the three muscles (equivalent to < 3% of the muscle length^49,50^), suggesting that the length-dependence of muscle strength should make limited effect on the MVIVS measured in the present study. On the other hand, the effect of the velocity-dependence of muscle strength was found substantial. Based on the measured elbow joint motion during pitching, the elbow flexor-pronator muscles should be in an eccentric condition and, therefore, the determined muscular varus strength in an isometric condition is likely to be an underestimation of the corresponding strength in dynamic condition in pitching delivery. According to a study on the specific tension (the maximum force development per unit physiological cross-sectional area) of elbow flexor and extensor muscles^51^, the elbow flexors increase the specific tension by 18% on average (22% for extensors) under an isokinetic eccentric condition in comparison to isometric condition. Another in-vivo study of isokinetic strength of elbow muscles also reported that the muscle’s force generation capability increased by 10% relative to an isometric condition^52^. These findings suggest that participants’ muscular varus strength in the pitching condition might be 78–84 N m for Sub1 and 65–70 N m for Sub2. With the estimated maximum voluntary eccentric varus strength, the ratio of the muscle torque required to resist the valgus load completely to the maximum varus strength decreases from 73.1–91.9%MVIVS to 61.9–83.5% of the estimated maximum eccentric strength. The numerical discussion here indicates clearly that the muscle varus strength measured in an isometric condition at an elbow flexion angle of 90° is likely to be an underestimation of the maximum varus torque in the actual contraction condition of the involved muscles during pitching delivery and that the computed %MVIVS value is likely to overestimate the corresponding percentage of varus strength in the actual contraction condition at the time of pitching. The systematic error associated with the velocity-dependence of muscle strength should always be taken into account when utilizing analytical results for risk assessment of baseball pitchers and planning of conditioning/rehabilitation programs.

## Conclusion

The present method successfully assessed if baseball pitchers have sufficient muscular varus strength to withstand the valgus loading and how strongly the elbow musculature must contract to reduce the loading on the UCL to fall within safe range. The method is expected to offer new insight to identify biomechanical risk of UCL injury in baseball pitchers.

## Data Availability

The datasets generated during and/or analyzed during the current study are available from the corresponding author on reasonable request.
